# Circadian Rhythms Tied to Changes in Brain Morphology in a Densely Sampled Male

**DOI:** 10.1523/JNEUROSCI.0573-24.2024

**Published:** 2024-08-15

**Authors:** Elle M. Murata, Laura Pritschet, Hannah Grotzinger, Caitlin M. Taylor, Emily G. Jacobs

**Affiliations:** ^1^Department of Psychological & Brain Sciences, University of California, Santa Barbara, Santa Barbara, California 93106; ^2^Neuroscience Research Institute, University of California, Santa Barbara, Santa Barbara, California 93106

**Keywords:** brain structure, diurnal rhythms, MRI, precision imaging, steroid hormones

## Abstract

Circadian, infradian, and seasonal changes in steroid hormone secretion have been tied to changes in brain volume in several mammalian species. However, the relationship between circadian changes in steroid hormone production and rhythmic changes in brain morphology in humans is largely unknown. Here, we examined the relationship between diurnal fluctuations in steroid hormones and multiscale brain morphology in a precision imaging study of a male who completed 40 MRI and serological assessments at 7 A.M. and 8 P.M. over the course of a month, targeting hormone concentrations at their peak and nadir. Diurnal fluctuations in steroid hormones were tied to pronounced changes in global and regional brain morphology. From morning to evening, total brain volume, gray matter volume, and cortical thickness decreased, coincident with decreases in steroid hormone concentrations (testosterone, estradiol, and cortisol). In parallel, cerebrospinal fluid and ventricle size increased from A.M. to P.M. Global changes were driven by decreases within the occipital and parietal cortices. These findings highlight natural rhythms in brain morphology that keep time with the diurnal ebb and flow of steroid hormones.

## Significance Statement

Though rhythmic changes in steroid hormone secretion have been tied to changes in brain volume in several mammalian species, this relationship has not been well-characterized in humans. In this precision neuroimaging study, we found that global and regional brain morphology and steroid hormone levels exhibit tandem circadian rhythms. These findings provide high-resolution insight into the anatomical signature of diurnal changes in brain morphology and steroid hormone production in a human male and reveal the metronomic regularity of these rhythms over time.

## Introduction

Endogenous biological rhythms guide physiological processes throughout the body in nearly all organisms. Some of the most pronounced biological rhythms are the time-varying secretion of steroid hormones. Testosterone, estradiol, and cortisol exhibit circadian rhythms, with production peaking in the morning and decreasing throughout the day (Rose et al., 1972; Barberia et al., 1973; Berg and Wynne-Edwards, 2001; Diver et al., 2003; Ankarberg-Lindgren and Norjavaara, 2008), yet the influence of this diurnal ebb and flow of hormone production on the human brain is remarkably understudied. Several lines of evidence suggest such a link exists. Rhythmic changes in steroid hormone secretion, often marked by increased levels in breeding season and reduced levels in winter (Prendergast et al., 2002), have been tied to changes in brain morphology in animal models. In voles, brain and hippocampal mass are elevated during the summer and reduced in winter months (Yaskin, 1984). Mice exhibit decreased total brain size and hippocampal volume (Pyter et al., 2005), and hamsters show reduced soma size in the hippocampus (Workman et al., 2011) in short versus long photoperiods. Songbirds also exhibit seasonal fluctuations in brain structure, morphological changes that coincide with changes in steroid hormone production (DeVoogd and Nottebohm, 1981; Nottebohm, 1981; Bottjer et al., 1986). Human MRI studies have begun to characterize the role of circadian rhythms in modulating brain structure and function. There are reports of diurnal differences in functional connectivity (Orban et al., 2020; Farahani et al., 2021), cerebral blood flow (Elvsåshagen et al., 2019), water diffusivity (Thomas et al., 2018; Tuura et al., 2021), and brain volume (Trefler et al., 2016; Karch et al., 2019; Zareba et al., 2023, 2024). However, the association between circadian changes in hormone production and rhythmic changes in the brain is unclear.

Over the past decade, the field of neuroimaging has recognized the need to go beyond studying group-level effects, in favor of study designs that track dynamic brain changes in individuals over time (Poldrack et al., 2015; Braga and Buckner, 2017; Gordon et al., 2017; Elliott et al., 2023; Reznik et al., 2023). This approach, often referred to as “dense sampling,” is particularly well-suited for exploring how the central nervous system interacts with the endocrine system. Indeed, precision neuroimaging studies have begun to advance the study of brain–hormone relationships by densely scanning individuals with high temporal resolution over periods of normative hormonal change (Pritschet et al., 2021; Jacobs, 2023; Pritschet and Jacobs, 2023). For example, our group found that rhythmic changes in steroid hormones across the 24 h diurnal cycle (Grotzinger et al., 2024) and 30 d menstrual cycle (Fitzgerald et al., 2020; Pritschet et al., 2020; Mueller et al., 2021) are tied to changes in the brain's functional network architecture. Changes in brain morphology are also evident across these timescales. Subregions of the medial temporal lobe wax and wane in step with endocrine changes across the menstrual cycle (Zsido et al., 2023), an effect that is eliminated by chronic hormone suppression (Taylor et al., 2020). Precision imaging studies are yielding new insights into steroid hormones as rapid modulators of brain plasticity, in keeping with 30 years of evidence from animal models (Taxier et al., 2020). To date, we lack a comparable understanding of how rhythmic endocrine changes influence brain morphology across shorter cycles, such as the diurnal change in hormone production.

To determine the relationship between diurnal hormone fluctuations and brain morphology, we used a dense sampling design to collect MRI, serum, saliva, and mood data from a healthy adult male every 12–24 h for 30 consecutive days. Time-locked sessions occurred at 7 A.M. and 8 P.M., capturing the peak and nadir of steroid hormone production. By metronomically collecting MRI and biofluid data, we were able to track the diurnal ebb and flow of endocrine and neural changes in the human brain.

Diurnal fluctuations in steroid hormones were tied to pronounced changes in global and regional brain morphology. From morning to evening, total measures of brain volume, cortical gray matter volume (GMV), and cortical thickness (CT) decreased significantly, paralleling decreases in testosterone, estradiol, and cortisol. Cerebrospinal fluid (CSF) volume and ventricle size showed the opposite pattern, increasing throughout the day. At the regional level, cortical reductions were most apparent in visual cortices, including the striate and extrastriate cortex; subcortical reductions were most evident in the cerebellum, brainstem, and right hippocampus. These findings demonstrate a natural rhythm to brain morphology, a cadence that pulses in time with the diurnal ebb and flow of steroid hormones.

## Materials and Methods

### Participant

The participant was a healthy 26-year-old right-handed Caucasian male with no history of neuropsychiatric diagnosis, endocrine disorders, major medical conditions, or prior head trauma. The participant was not taking any prescription medication nor did he experience any transitory diseases prior to or for the duration of the study. The participant gave written informed consent for a study approved by the University of California, Santa Barbara Human Subjects Committee and was paid for participation in the study.

### Experimental design

**Figure 1.**
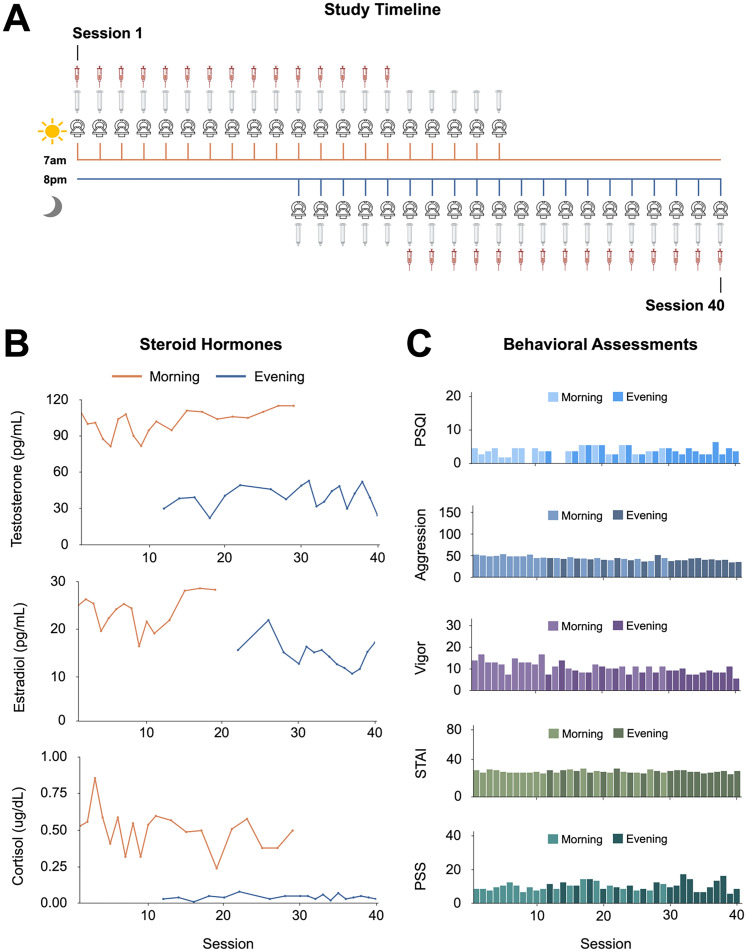
Precision imaging study design. ***A***, For 30 consecutive days, a healthy 26-year-old male underwent brain imaging and serological assessment every 12–24 h for a total of 40 sessions. ***B***, Steroid hormones demonstrated typical diurnal patterns, with testosterone (saliva), estradiol (only assessed with serum), and cortisol (saliva) peaking in the morning and significantly reduced in the evening. Note that saliva sampling was performed at each session (testosterone, cortisol; *n* = 40); a blood sample was obtained once per day (estradiol; *n* = 30), following human subjects protocol regulations. ***C***, The subject completed mood assessments (e.g., sleep quality, mood, anxiety, stress) at each session. All measures were within the standard range and remained consistent across the study duration. Abbreviations: PSQI, Pittsburgh Sleep Quality Index; STAI, State-Trait Anxiety Inventory; PSS, Perceived Stress Scale. Steroid hormone concentrations by time of day are reported in Extended Data Table 1-1.

The methods for this study are reported in Grotzinger et al. (2024). Briefly, the participant underwent venipuncture and brain imaging every 12–24 h for 30 consecutive days. At each session, the participant completed a questionnaire to assess stress, sleep, and mood states, followed by endocrine sampling at 7 A.M. (morning sessions) and/or at 8 P.M. (evening sessions). The participant gave a ∼2 ml saliva sample at each session, followed by a blood sample. On days with morning and evening sessions, the participant underwent one blood draw per day (Fig. 1*A*), per safety guidelines. Morning endocrine samples were collected after at least 8 h of overnight fasting, and evening endocrine samples were collected following 1.5 h of abstaining from consumption of food or drink (excluding water). The participant refrained from consuming caffeinated beverages before each morning session.

10.1523/JNEUROSCI.0573-24.2024.t1-1 Table 1-1 Steroid hormone concentrations by time of day. Download Table 1-1, DOCX file.

10.1523/JNEUROSCI.0573-24.2024.t1-2 Table 1-2 Correlations between gray matter volume in select cortical regions and steroid hormones. Download Table 1-2, DOCX file.

10.1523/JNEUROSCI.0573-24.2024.t1-3 Table 1-3 Correlations between cortical thickness in cortical regions and steroid hormones. Download Table 1-3, DOCX file.

#### Behavioral assessments

The following scales (adapted to reflect the past 12–24 h) were administered at each session as part of the questionnaire: the Perceived Stress Scale (Cohen et al., 1983), Pittsburgh Sleep Quality Index (PSQI; Buysse et al., 1989), State-Trait Anxiety Inventory for Adults (Speilberger and Vagg, 1984), Profile of Mood States (Pollock et al., 1979), and Aggression Questionnaire (Buss and Perry, 1992). The questionnaire for the evening sessions excluded the PSQI to avoid redundancy. All mood measures fell within standard reference ranges (Fig. 1*C*).

#### Endocrine procedures

A ∼2 ml saliva sample was obtained via passive drooling into a wide-mouthed plastic cryovial. The participant refrained from eating and drinking (besides water) at least 1.5 h before saliva sample collection, and the morning samples were collected after fasting overnight. The participant pooled saliva for 5–10 min before depositing the sample into the cryovial to determine total testosterone and cortisol concentrations. The sample was stored at −20°C until assayed. Saliva concentrations were determined via enzyme immunoassay at Brigham and Women's Hospital Research Assay Core. Immediately after the saliva sample was obtained, a licensed phlebotomist inserted a saline-lock intravenous line into either the dominant or nondominant hand or forearm daily to evaluate total testosterone, cortisol, and 17β-estradiol concentrations. One 10cc ml blood sample was collected in a vacutainer SST (BD Diagnostic Systems) each session. The sample clotted at room temperature for ∼60  min until centrifugation (2,100 × *g* for 10  min) and was then aliquoted into three 2 ml microtubes. Serum samples were stored at −20°C until assayed. Serum concentrations were determined via liquid chromatography mass spectrometry at the Brigham and Women's Hospital Research Assay Core. Assay sensitivities, dynamic range, and intra-assay coefficients of variation (respectively) were as follows: estradiol, 1 pg/ml, 1–500 pg/ml, <5% relative standard deviation (RSD); testosterone, 1.0 ng/dl, 1–200  ng/dl, <2% RSD; cortisol, 0.5  ng/ml, 0.5–250 pg/ml, <8% RSD. Note that estradiol measurements were acquired from serum samples, resulting in 30 timepoints.

### MRI acquisition

At each session, the participant underwent a whole-brain structural MRI and 15 min eyes-open resting-state scan conducted on a Siemens 3 T Prisma scanner equipped with a 64-channel phased-array head coil. High-resolution anatomical scans were acquired using a T1-weighted magnetization-prepared rapid gradient echo (MPRAGE) sequence (TR, 2,500 ms; TE, 2.31 ms; TI, 934 ms; flip angle, 7°; 0.8 mm thickness), followed by a gradient echo fieldmap (TR, 758 ms; TE_1_, 4.92 ms; TE_2_, 7.38 ms; flip angle, 60°). A T2-weighted turbo spin echo (TSE) scan was also acquired with an oblique coronal orientation positioned orthogonally to the main axis of the hippocampus (TR/TE, 8,100/50 ms; flip angle, 122°; 0.4 × 0.4 mm^2^ in-plane resolution; 2 mm slice thickness; 31 interleaved slices with no gap; total acquisition time, 4:21 min). Resting-state functional MRI scans allowed for indirect assessments of head motion (Grotzinger et al., 2024). A quantitative assessment of structural image quality (i.e., FreeSurfer-derived Euler number; Rosen et al., 2018) indicated no significant difference between morning and evening sessions (*p *= 0.12).

### Whole-brain morphology assessments

Image processing for evaluating GMV, white matter volume (WMV), and CT was conducted with Advanced Normalization Tools (ANTs, version 2.1.0). A subject-specific anatomical template (SST) was created with multivariate template construction. Subject-specific tissue priors were created using the OASIS population template priors from ANTs. Structural images from each session were then registered to this template and processed with the ANTs CT pipeline (Tustison et al., 2014). This pipeline consists of N4 bias correction, tissue segmentation, and cortical thickness estimation. N4 bias correction minimizes field inhomogeneity effects. Tissue segmentation via Atropos makes tissue segmentation estimates of white matter, gray matter, deep gray matter, and cerebrospinal fluid by using prior knowledge to guide segmentation. The Diffeomorphic Registration-based Cortical Thickness (DiReCT) algorithm was used to evaluate CT. CT measures were created based on diffeomorphic mappings between gray matter–white matter interface and the estimated gray matter–cerebrospinal fluid interface (Das et al., 2009). Gray matter measures were created by first normalizing each gray matter tissue mask to the template. Then, the mask was multiplied to a Jacobian image computed by affine and nonlinear transforms. Brain volume and CT summary measures were output automatically for each scan session. Total brain volume, the sum of all voxels in the brain, is an estimate of the total intracranial volume. The brain was parcellated based on the 400-node Schaefer cortical atlas (Schaefer et al., 2018) in order to compare regions with those impacted at the functional level in the same participant (Grotzinger et al., 2024). This parcellation was first warped to the SST then applied to volumetric images to assess regional-level measures of CT and GMV by extracting the first eigenvariate across all voxels within each region. Regional-level metrics were computed by averaging across nodes into 41 broad regions (e.g., Extrastriate Cortex, Frontal Eye Fields—see Extended Data Table 3-1 for all regions). Measures of CSF, ventricle size, and subcortical volume were calculated by running the T1w images through the longitudinal recon-all pipeline from FreeSurfer (Dale et al., 1999; Reuter et al., 2012) and were segmented into 28 regions-of-interest determined via the aseg parcellation (Fischl et al., 2002).

### Medial temporal lobe subfield assessments

High-resolution medial temporal lobe volumes (CA1, CA2/3, dentate gyrus, subiculum, perirhinal, entorhinal and parahippocampal cortex) were measured from a dedicated T2-weighted image of the hippocampi, coregistered to a T1-weighted MRI using the automatic segmentation of hippocampal subfields (ASHS) package (Yushkevich et al., 2015) and the Princeton Young Adult 3T ASHS Atlas (*n* = 24; mean age, 22.5 years; Aly and Turk-Browne, 2015). T2w scans and segmentations were first visually examined using ITK-SNAP (Yushkevich et al., 2006) for quality assurance and then subjected to manual editing in native space using ITK-SNAP (v.3.8.0-b; author CMT). Boundaries between perirhinal, entorhinal, and parahippocampal cortices were established in keeping with the Olsen–Amaral–Palombo (OAP) segmentation protocol (Palombo et al., 2013). In instances where automatic segmentation did not clearly correspond to the underlying neuroanatomy, such as when a certain label was missing several gray matter voxels, manual retouching allowed for individual voxels to be added or removed (Extended Data Fig. 4-3). All results are reported using the manually retouched subregion volumes to ensure the most faithful representation of the underlying neuroanatomy. Scans were randomized and segmentation was performed in a random order. Medial temporal lobe subfield volumes were averaged across hemispheres.

### Statistical analyses

All analyses were conducted in R (version 4.3.2). Independent samples *t* tests were run to compare global and regional brain structure measures between morning and evening sessions. *t* test results were considered statistically significant if they met Bonferroni’s correction for multiple comparisons: *p *< 0.006 (*p *< 0.05/9 measures) for global morphology measures; *p *< 0.007 (*p *< 0.05/7 regions) for medial temporal subfields; *p *< 0.001 (*p *< 0.05/41 regions) for regional cortical measures; and *p *< 0.002 (*p *< 0.05/28 regions) for regional subcortical measures. Effect size was estimated using Cohen's *d*. To evaluate the impact of time of day (TOD) on GMV and CT in individual nodes, multivariate regressions were performed and false discovery rate (FDR) corrected. Additionally, correlation matrices between brain measures and steroid hormones [testosterone (saliva), estradiol (serum), cortisol (saliva)] were computed and FDR corrected. Because most steroid hormones (testosterone, cortisol) did not display a normal distribution (Shapiro–Wilk test: *p *< 0.05), nonparametric Spearman's rank correlations were computed. To note, session 24 was excluded from analyses due to an abnormal dip in testosterone after the participant received the COVID-19 vaccine. In this evening session, total testosterone (saliva) was 6.54 pg/ml, which was ∼83% lower than the average evening testosterone level (*M* = 37.93 pg/ml).

## Results

Evaluations of steroid hormones confirmed the expected peak of testosterone, estradiol, and cortisol in the morning and nadir in the evening. Testosterone, estradiol, and cortisol decreased from morning to evening by ∼61, ∼38, and ∼92%, respectively (Fig. 1*B*, Extended Data Table 1-1).

### Global brain morphology by time of day and steroid hormone levels

**Figure 2.**
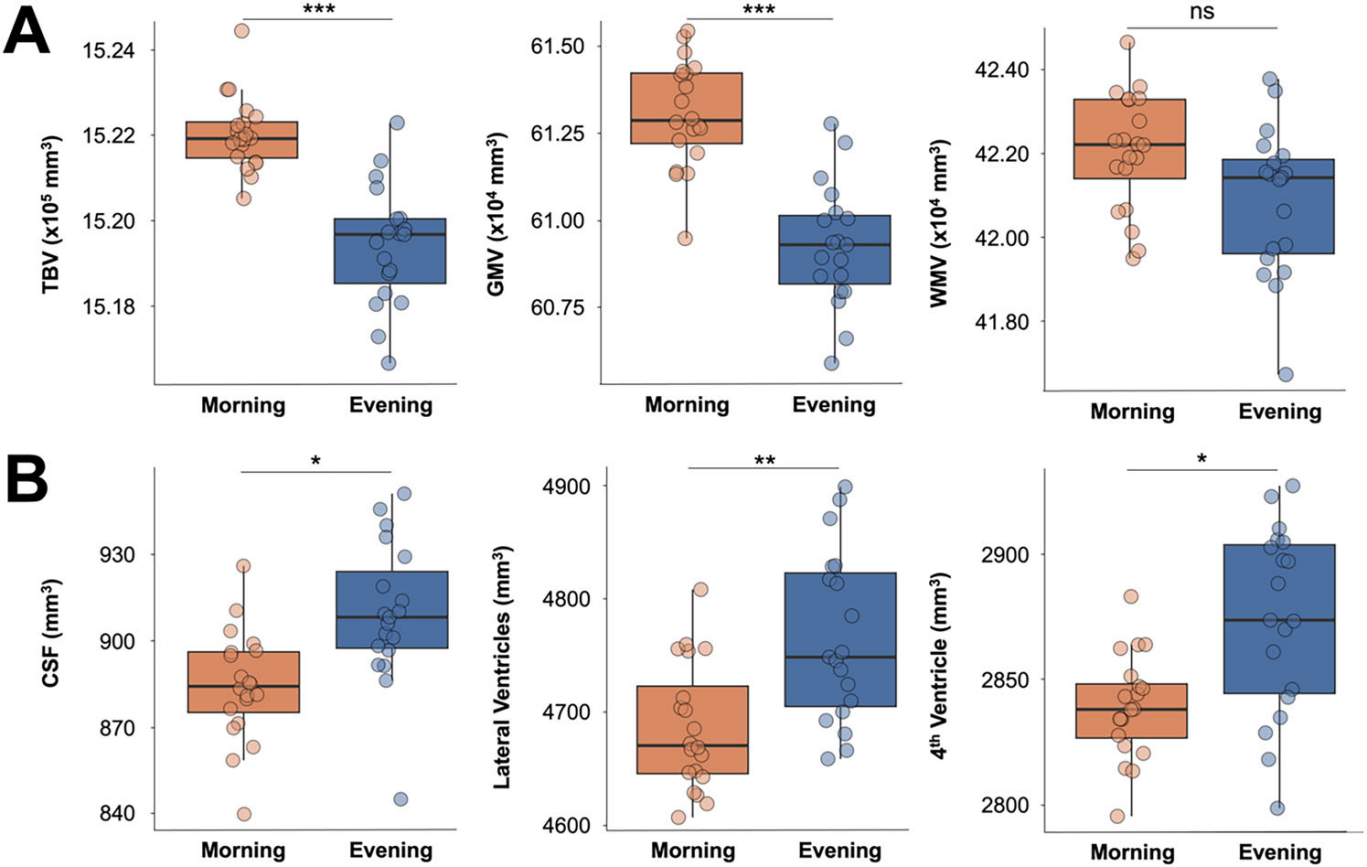
Neuroanatomical changes throughout the day in a densely sampled male. ***A***, Total brain volume and gray matter volume decreased over the course of the day, while white matter volume did not (at a Bonferroni-corrected threshold). ***B***, Cerebrospinal fluid, lateral ventricle, and fourth ventricle volume all increased from morning to evening. Abbreviations: TBV, total brain volume; GMV, gray matter volume; WMV, white matter volume; CSF, cerebrospinal fluid; ns, not significant; *p < 0.006, **p < 0.001, ***p < 0.0001. Hormone and behavior correlations with brain morphology measures are reported in Extended Data Table 2-1 and Extended Data Figure 2-1, respectively.

Changes in brain morphology were evident by TOD. Total brain volume (*t*_(29.37)_ = −6.93; *p *< 0.001; *d *= −2.23) and total GMV (*t*_(35.70)_ = −7.22; *p *< 0.001; *d *= −2.32) peaked in the morning and dipped in the evening. There was no observable difference in WMV at a corrected significance threshold (*t*_(34.33)_ = −2.32; *p *= 0.026; *d *= −0.75; Fig. 2*A*, Extended Data Table 2-1). In contrast, global increases were observed in CSF (*t*_(34.00)_ = 3.53; *p *= 0.001; *d *= 1.14), lateral ventricles (*t*_(33.27)_ = 3.72; *p *< 0.001; *d *= 1.97), and fourth ventricle (*t*_(27.68)_ = 3.60; *p *= 0.001; *d *= 1.16) volumes from morning to evening (Fig. 2*B*, Extended Data Table 2-1). With the exception of WMV, Cohen's *d* values indicated a large effect of TOD on these global brain morphology measures. These relationships paralleled diurnal changes in steroid hormone concentrations. Steroid hormones demonstrated a positive correlation with total brain volume (testosterone: *r *= 0.58, *p *< 0.001; estradiol: *r *= 0.70, *p *< 0.001; cortisol: *r *= 0.56, *p *< 0.001) and global GMV (testosterone: *r *= 0.67, *p *< 0.001; estradiol: *r *= 0.69, *p *< 0.001; cortisol: *r *= 0.61, *p *< 0.001). Global cortical thickness demonstrated a comparable decrease from morning to evening, with the same positive relationship with all three hormones (Extended Data Table 2-1). With the exception of aggression and vigor, there were no significant associations between global brain morphology measures and mood assessments (Extended Data Fig. 2-1). Though total brain volume (estimated intracranial volume) demonstrated a diurnal rhythm, it fluctuated by ∼0.17%, which is ∼3–16-fold smaller than GMV, CSF, lateral ventricle, and cortical thickness changes.

10.1523/JNEUROSCI.0573-24.2024.t2-1 Table 2-1 Global brain morphology by time of day and association with steroid hormones. Download Table 2-1, DOCX file.

10.1523/JNEUROSCI.0573-24.2024.f2-1 Figure 2-1 **Correlations between global brain morphology and behavioral assessments.** Correlation plot shows relationships between global brain morphology measures and mood assessments. Cool colors indicate positive correlations and warm colors indicate negative correlations. FDR-corrected at *q *< .05: *p < .05, **p < .01, ***p < .001Abbreviations: GMV = Gray Matter Volume; WMV = White Matter Volume; TBV = Total Brain Volume; CT = Cortical Thickness, CSF = Cerebrospinal Fluid; PSQI = Pittsburgh Sleep Quality Index; STAI = State-Trait Anxiety Inventory; PSS = Perceived Stress Scale. Download Figure 2-1, TIF file.

### Regional gray matter volume is tied to time of day and steroid hormone concentrations

**Figure 3.**
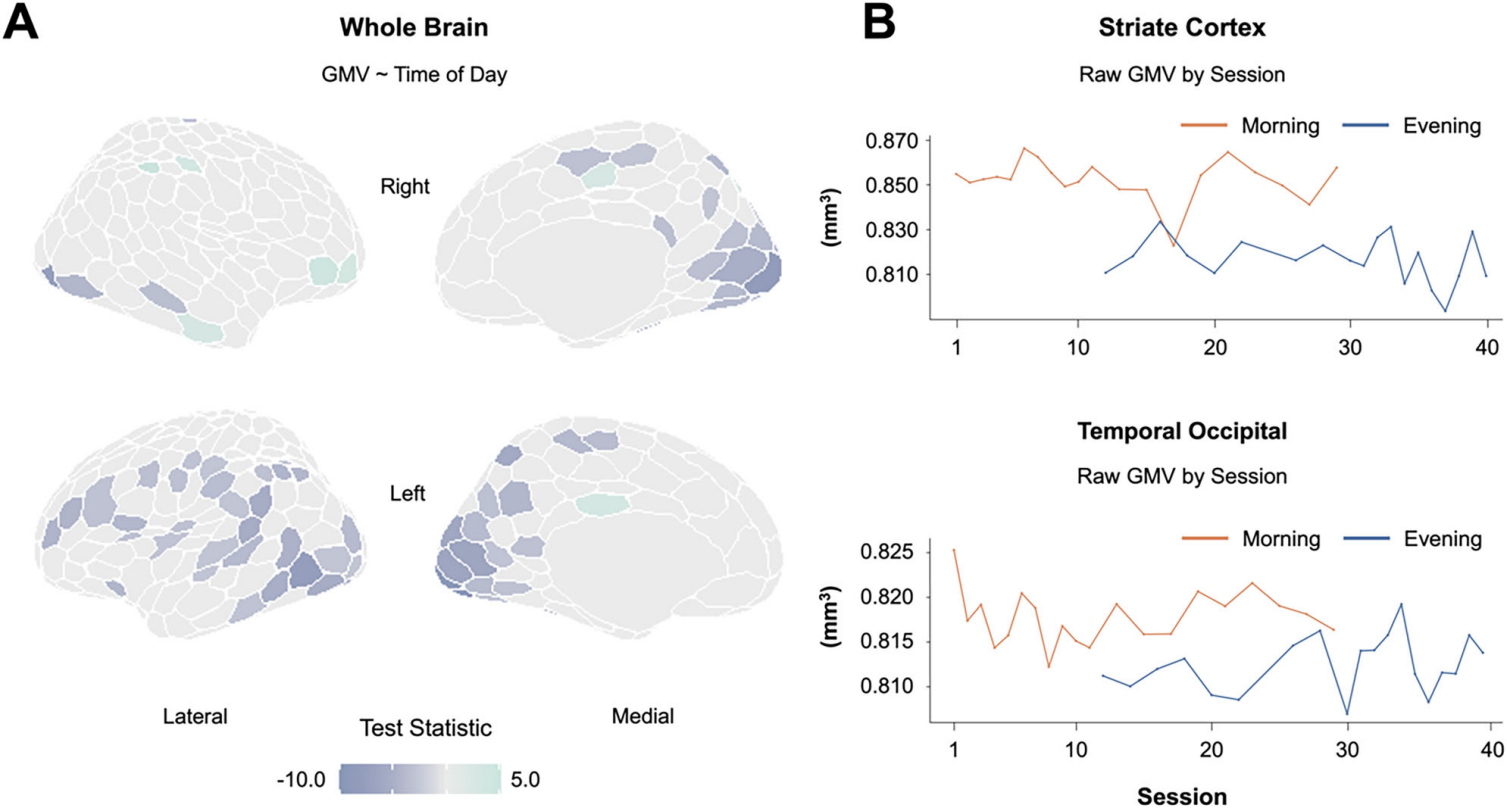
Regional neuroanatomical changes between morning and evening sessions. ***A***, Multivariate regression demonstrates a mostly negative relationship between time of day and gray matter volume (FDR at *q *< 0.05; nonsignificant test statistics were set to zero for interpretability). ***B***, Gray matter volume in the striate cortex and temporal occipital regions are heightened in the morning and reduced in the evening. Abbreviations: GMV, gray matter volume. For additional regions, see Extended Data Table 3-1 and for regional cortical thickness changes, see Extended Data Table 3-2.

To investigate whether changes in morphology were ubiquitous throughout the brain, a regional approach was taken to pinpoint areas most sensitive to diurnal rhythms. Pronounced diurnal decreases in GMV were evident in the parietal and occipital cortices, including extrastriate and striate, temporal occipital, and precuneus regions. The effect size was large across regions (all *d < *−1.15; *p *< 0.001; Fig. 3, Extended Data Table 3-1). The magnitude of these regional reductions was strongly associated with the diurnal reduction in steroid hormones (Table 1, Extended Data Table 1-2). Global changes in volume appear to be driven by these occipital and parietal regions, as the majority of other regions were largely stable from A.M. to P.M. (Extended Data Table 3-1). Cortical thickness demonstrated the same pattern (Extended Data Table 3-2 and Extended Data Table 1-3).

10.1523/JNEUROSCI.0573-24.2024.t3-1 Table 3-1 Gray matter volume in cortical regions by time of day. Download Table 3-1, DOCX file.

10.1523/JNEUROSCI.0573-24.2024.t3-2 Table 3-2 Cortical thickness in cortical regions by time of day. Download Table 3-2, DOCX file.

**Table 1.** Correlations between GMV in select cortical regions and steroid hormones

	Correlation		
Brain region	Testosterone (saliva)	Estradiol (serum)	Cortisol (saliva)
Extrastriate cortex	0.62***	0.63**	0.67***
Striate cortex	0.71***	0.69***	0.80***
Extrastriate inferior	0.28	0.46*	0.31
Striate calcarine	0.51**	0.71***	0.65***
Extrastriate superior	0.52**	0.52*	0.51**
Somatomotor	0.23	0.20	0.23
Auditory	0.26	0.17	0.24
Insula	0.34	0.23	0.20
Secondary somatomotor	0.16	0.11	0.11
Central	0.16	0.07	0.20
Temporal occipital	0.65***	0.63**	0.54**
Parietal occipital	0.43*	0.31	0.36
Superior parietal lobule	5.77 × 10^−3^	−0.21	−0.30
Post central	0.16	0.01	8.12 × 10^−3^
Frontal eye fields	0.38	0.22	0.22
Orbitofrontal cortex	0.06	0.11	−0.10
Lateral dorsal PFC	0.34	0.33	0.37
Mid-cingulate	0.02	−0.06	0.09
Inferior parietal lobule	0.34	0.29	0.21
Precuneus	0.31	0.33	0.32
Precentral ventral	0.36	0.46*	0.32
Lateral PFC	0.39*	0.43	0.40*

Several brain regions, including the extrastriate cortex, striate cortex, striate calcarine, and temporal occipital, were positively correlated with steroid hormone concentrations (testosterone, estradiol, and cortisol). For the full list of cortical correlation results, see Extended Data Table 1**-**2. For cortical thickness correlations, see Extended Data Table 1-3. FDR corrected at *q *< 0.05. Testosterone, pg/ml; estradiol, pg/ml; cortisol, µg/dl. GMV, gray matter volume; PFC, prefrontal cortex.

* *p *< 0.05, **p < 0.01, ***p < 0.001.

### Diurnal subcortical volume reduction is regionally specific

**Figure 4.**
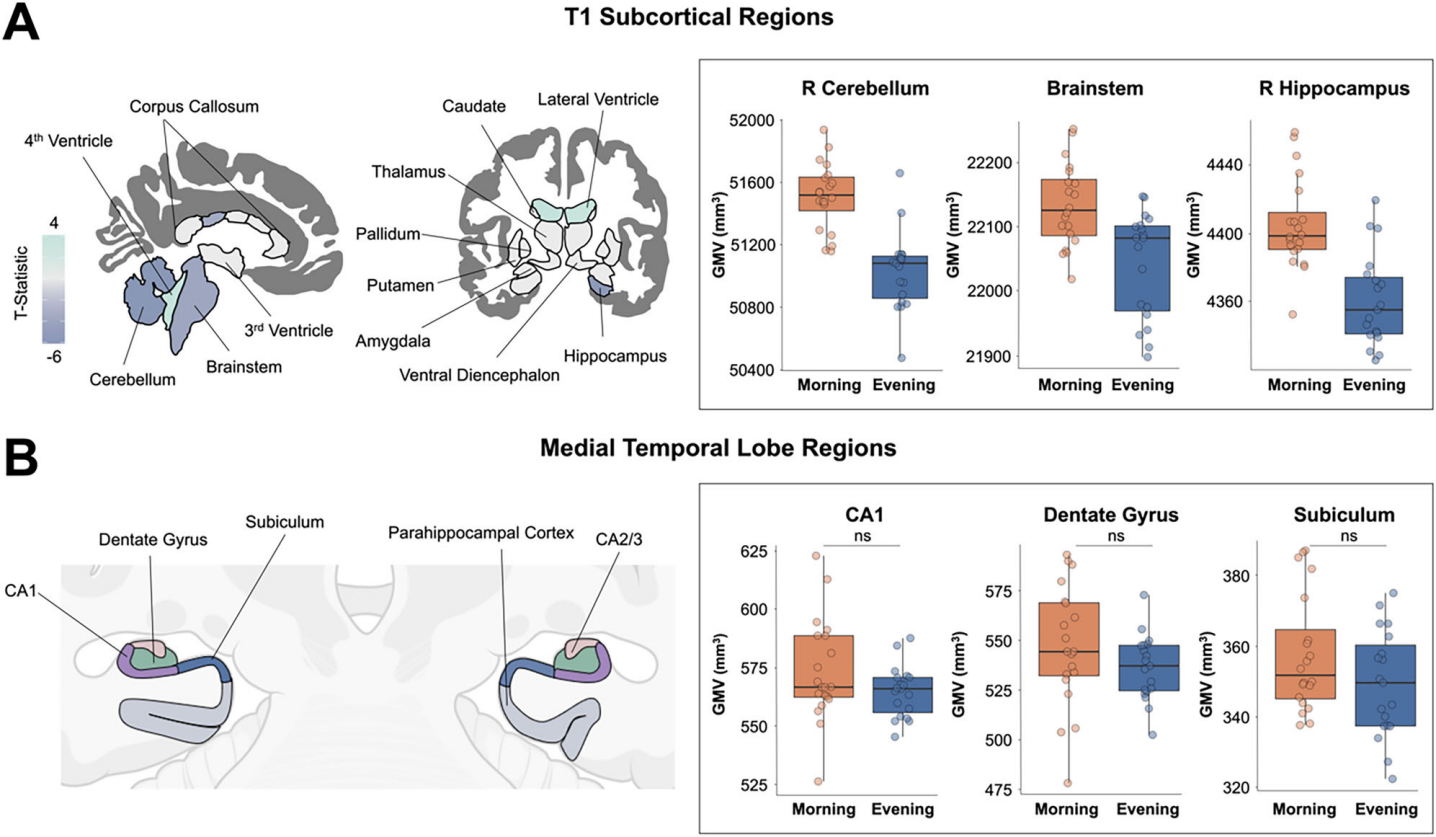
Subcortical gray matter volume by time of day. ***A***, Multivariate regression for subcortical regions demonstrated decreasing volume throughout the day. Left, Purple tones indicate volumetric reduction while aqua tones indicate volumetric expansion from A.M. to P.M. (FDR at *q *< 0.05; nonsignificant test statistics were set to zero for interpretability). Gray matter volume was higher in the right cerebellum, brainstem, and right hippocampus in morning compared with evening sessions (see example box plots on the right). For the full list of subcortical results, see Extended Data Table 4-1. ***B***, Diagram illustrates medial temporal lobe subregion segmentation from the ASHS parcellation. Gray matter volume in CA1, dentate gyrus, and subiculum did not change throughout the day. This pattern held for all medial temporal lobe subregions (CA2/3, entorhinal cortex, perirhinal cortex, and parahippocampal cortex, *p *> 0.007 for all, Extended Data Table 4-2). For a manual segmentation example, see Extended Data Figure 4-3. Abbreviations: GMV, gray matter volume; ns, not significant.

Total right hippocampal volume decreased significantly from morning to evening (*t*_(36.89)_ = −5.17; *p *< 0.001). Similarly, right and left cerebellum and brainstem volumes were significantly reduced from morning to evening scans (*t*_(35.59)_ = −6.41, *p *< 0.001; *t*_(36.27)_ = −8.38, *p *< 0.001; and *t*_(34.06)_ = −3.87, *p *< 0.001, respectively). The effect size was large across these regions (all *d *< −1.24), and they demonstrated positive correlations with steroid hormones (Fig. 4*A*, Extended Data Table 4-1). When assessing subfield volumes of the hippocampal body, we observed no changes by TOD. All seven subfield volumes remained stable throughout the day (Bonferroni’s corrected *p *> 0.007), and there were no significant relationships between subfield volumes and steroid hormones (Fig. 4*B*, Extended Data Table 4-2).

10.1523/JNEUROSCI.0573-24.2024.t4-1 Table 4-1 Subcortical gray matter volume by time of day and association with steroid hormones. Download Table 4-1, DOCX file.

10.1523/JNEUROSCI.0573-24.2024.t4-2 Table 4-2 Medial temporal lobe gray matter volume by time of day and association with steroid hormones. Download Table 4-2, DOCX file.

10.1523/JNEUROSCI.0573-24.2024.f4-3 Figure 4-3 **Manual segmentation of medial temporal lobe. Example of manual retouching.** A) Sample original ASHS segmentation with erroneous labeling of CA2/3 within dentate gyrus, missing/unlabeled voxels within the subiculum, and inclusion of CSF in PHC label. B) Segmentation of CA2/3, subiculum and PHC after manual retouching. Download Figure 4-3, TIF file.

## Discussion

Here we investigated the impact of circadian rhythms on global and regional brain morphology in a densely sampled male. Total brain volume, GMV, and CT decreased from morning to evening, while CSF and ventricle size increased. These changes were not uniform across the cortical mantle, rather driven by A.M. to P.M. decreases in GMV and CT in occipital and parietal cortices. These results build on a growing literature demonstrating the robust influence of biological rhythms on human brain morphology.

The direction and magnitude of the observed changes in global brain morphology are consistent with existing MRI studies of TOD. Previous reports suggest a decrease in GMV (∼2% of ICV; Trefler et al., 2016) and ∼1% decrease in both GMV and cortex volume (Karch et al., 2019) over the course of a day in healthy populations. Here, we observed a ∼0.62% decrease in GMV on average from morning to evening.

The most prominent changes in brain morphology were evident in parietal and occipital cortices, which showed decreases in both GMV and CT from morning to night. These changes were strongly correlated with steroid hormone levels, including testosterone, estradiol, and cortisol. Our results are in keeping with a recent study exploring the effect of diurnal rhythms on brain structure, where GMV and CT in the primary visual cortex were tied to diurnal variation in mood and cognition (Zareba et al., 2023). The volumetric changes in posterior cortices are mirrored by changes in brain function. In a parallel analysis, our group examined changes in functional connectivity from A.M. to P.M. in the same densely sampled male. Connectivity in visual networks were particularly sensitive to TOD. Nodes belonging to central and peripheral visual networks demonstrated some of the strongest associations with diurnal variation in testosterone, estradiol, and cortisol (Grotzinger et al., 2024), paralleling the changes in GMV and CT we see here. Hormonal regulation of visual cortex has been demonstrated in rodent studies. For example, ovarian hormone exposure led to altered cortical neuron number in the primary visual cortex (Nuñez et al., 2002), while androgen exposure prevented cell death in the visual cortex (Nuñez et al., 2000). One human study demonstrated TOD effects on GMV outside of the parietal and occipital cortices. The authors scanned individuals once between 10 A.M. and 12 P.M. and again between 2 P.M. and 4 P.M. and reported changes in the frontal and temporal lobe (Trefler et al., 2016). This design was outside of the window aligned with the peak and trough of diurnal steroid hormone production, a difference from the present study that could be driving these regional discrepancies in cortical GMV findings.

We also determined the extent to which subcortical structures change by TOD, noting significant decreases in GMV in the cerebellum, brainstem, and right hippocampus. Next, we employed high-resolution imaging and segmentation of the hippocampal body to assess medial temporal lobe subregions (including dentate gyrus, CA1, CA2/3, subiculum, parahippocampal cortex, entorhinal cortex, and perirhinal cortex), revealing stability over time. The discrepancy between whole hippocampal volume and subfield-level volumes within the hippocampal body suggest that the head and tail of the hippocampus may be the loci of diurnal change, though future work needs to be done to clarify these differences. Existing studies on TOD-related subcortical changes are mixed. Shorter photoperiods have been associated with smaller total hippocampal volumes (Miller et al., 2015) and smaller brainstem volumes (Majrashi et al., 2020). In contrast, Zareba et al. (2024) reported an increase in GMV in the amygdala and hippocampus from morning to evening. The present study, which tracks brain and hormone dynamics at a high temporal resolution, suggests an overall decrease in cortical and subcortical GMV from morning to evening—a pattern that mirrors the diurnal decrease in steroid hormones.

While it is clear that brain morphology exhibits circadian change, the role of steroid hormones in shaping diurnal changes in brain volume is not definitive, as results from the present study are correlational. Restricting correlations of brain morphology with steroid hormones to morning or evening sessions separately yielded no significant relationships. However, biological rhythms over longer timescales establish steroid hormones’ role in shaping brain morphology. Across the ∼28 d menstrual cycle, endogenous fluctuations in progesterone are associated with changes in GMV in the CA2/3, parahippocampal, entorhinal, and perirhinal cortex regions (Taylor et al., 2020); disrupting this hormonal rhythm eliminates these GMV changes. Further, changes in progesterone are tied to cerebellar changes in functional brain network organization (Fitzgerald et al., 2020). Steroid hormone expression in the mammalian cerebellum (Pang et al., 2013) and hippocampus (Brinton et al., 2008) is well-established, underscoring the hormonal regulation of these regions. The present findings are consistent with a model in which steroid hormones contribute to morphological changes in these regions at shorter, circadian, timescales. A number of other basic physiological variables that vary across the day could also contribute to the observed results, such as consumption/hydration, alertness, respiratory rate, and caloric intake.

Brain volume reduction throughout the day may be related to diurnal regulation of metabolic and synaptic machinery via the glymphatic system. Through the glymphatic pathway, metabolic buildup during the day exits by the exchange of CSF with interstitial fluid. CSF enters the brain, mixes with interstitial fluid, and is removed from the brain through venous drainage (Hauglund et al., 2020). This process is mediated by aquaporins, channels that are a key pathway for water movement. Aquaporin subtype 4 (AQP4) is a water channel found in astrocytes that helps maintain water homeostasis in the brain (Badaut et al., 2002). Aquaporin activity may be under the control of circadian rhythms and steroid hormones. In mice, localization of AQP4 to the vasculature, and glymphatic influx, peaked during the daytime, and knocking out AQP4 abolished these day–night changes in glymphatic influx (Hablitz et al., 2020). Aquaporins have also been identified in the paraventricular nucleus of the hypothalamus (Jung et al., 1994), a structure that plays an essential role in driving circadian rhythms and steroid hormone production. Circadian patterns of glymphatic activity may be driven by steroid hormones, which modulate aquaporin activity. Testosterone administration increases AQP4 protein levels in astrocytes (Gu et al., 2003). In contrast, estradiol or progesterone treatment decreases AQP4 expression and brain water content (Soltani et al., 2016). A potential mechanism for steroid hormone-mediated brain changes could be that declining steroid hormones throughout the day coincides with increased AQP4 expression and increased CSF and ventricle volume. Since CSF and ventricle expansion or swelling may lead to reductions in brain volume, an aquaporin-mediated increase in fluid dynamics could explain the observed decline in brain volume throughout the day.

Why might these diurnal rhythms in the brain exist? For most species, behavior is heavily organized around a day/night cycle. Diurnal rhythms are ubiquitous in nature and have broad implications for animal cognition and behavior. The consequences of disrupting circadian rhythms are increasingly well-characterized in animal and human studies (Walker et al., 2020). Major depressive disorder, seasonal affective disorder, and bipolar disorder have all been tied to disruptions in circadian cycles (Dollish et al., 2024). Metabolic side effects, such as abnormal glucose metabolism and lower insulin sensitivity, are well-established consequences of circadian rhythm disruption (Potter et al., 2016), as are memory impairments (Smies et al., 2022; Bellfy et al., 2023). In short, biological rhythms in the brain likely regulate mood and cognition. In future precision imaging studies, tying cyclic changes in regional and global brain volume to changes in behavior warrants investigation.

This precision imaging study mapped neuroanatomical changes across the circadian cycle in a single male with unprecedented temporal resolution. This adds to a new class of brain imaging studies that are revealing the dynamic nature of the human brain only identifiable at the individual level, previously blurred with group-averaged data. A limitation inherent to precision imaging studies is the inability to generalize findings to a broader population. However, the diurnal rhythms we observed are consistent with findings reported in cross-sectional studies. Future precision imaging studies can expand on this work by sampling demographically diverse individuals, including those with disrupted circadian rhythms (e.g., individuals who work a night shift, flight attendants) and altered hormonal milieus (e.g., via pharmacological manipulation). Such studies would aid in disentangling the unique role of steroid hormones from other variables that may contribute to the rapid structural changes occurring in the brain diurnally. Such designs place a high demand on individuals volunteering to undergo frequent sampling. It should be noted that our participant's behavioral assessments of sleep, stress, and anxiety remained unchanged from the beginning to the end of our study (Fig. 1*C*), and researchers should keep track of such psychological factors when conducting extensive longitudinal protocols. These findings have substantial implications for the neuroimaging community: given the pronounced effects of diurnal rhythms on brain morphology, MRI experiments should adjust for TOD in all analyses.

Finally, hormonal fluctuations in females are often considered a source of unwanted variability (Beery and Zucker, 2011; Shansky, 2019), and this study suggests that steroid hormones are tied to aspects of brain morphology and function in males as well. Rhythmic production of steroid hormones should not be considered noise, but rather a foundational feature of biology.

## Data Availability

The dataset consists of 40 MRI scans (T1w, T2-hippoocampal, and resting-state fMRI scans) alongside state-dependent measures and serum assessments of steroid hormones for each session. These data are publicly available at openneuro.org/datasets/ds005115.
