# Supplementary material for: Circadian Rhythms Tied to Changes in Brain Morphology in a Densely Sampled Male
Source: J Neurosci. 2024 Aug 15;44(38):e0573242024. doi: 10.1523/JNEUROSCI.0573-24.2024 (PMC11411591; doi:10.1523/JNEUROSCI.0573-24.2024)
Supplement: Table 1-1 — Steroid hormone concentrations by time of day. Download Table 1-1, DOCX file. [file jneuro-44-e0573242024-s003.docx]

| Table 1-1. Steroid hormone concentration by time of day | | | |
| --- | --- | --- | --- |
|  | Morning  Mean (SD) | Evening  Mean (SD) | p-value |
| Testosterone (saliva) | 101.61 (10.24) | 39.58 (9.08) | 2.20e-16 |
| Estradiol (serum) | 23.77 (3.59) | 14.68 (2.86) | 4.45e-08 |
| Cortisol (saliva) | 0.50 (0.13) | 0.04 (0.02) | 2.89e-12 |
| Testosterone: pg/mL, Estradiol: pg/mL, Cortisol: ug/dL | | | |
