# Supplementary material for: Circadian Rhythms Tied to Changes in Brain Morphology in a Densely Sampled Male
Source: J Neurosci. 2024 Aug 15;44(38):e0573242024. doi: 10.1523/JNEUROSCI.0573-24.2024 (PMC11411591; doi:10.1523/JNEUROSCI.0573-24.2024)
Supplement: Table 1-2 — Correlations between gray matter volume in select cortical regions and steroid hormones. Download Table 1-2, DOCX file. [file jneuro-44-e0573242024-s006.docx]

| Table 1-2. Correlations between GMV in select cortical regions and steroid hormones | | | | |
| --- | --- | --- | --- | --- |
|  | Correlation | | | |
| Brain Region | | Testosterone  (saliva) | Estradiol  (serum) | Cortisol  (saliva) |
| Dorsal PFC | | 0.27 | 0.27 | 0.35 |
| Parietal Operculum | | 0.22 | 0.20 | 0.15 |
| Frontal Operculum | | 0.26 | 0.25 | 0.23 |
| Parietal Medial | | 0.21 | 0.02 | 0.15 |
| Precuneus | | 0.31 | 0.33 | 0.32 |
| Medial Posterior PFC | | 0.24 | 0.36 | 0.20 |
| Temporal Pole | | 0.15 | 0.18 | 0.22 |
| Temporal | | 0.18 | 0.15 | 0.03 |
| Intraparietal Sulcus | | 0.21 | 0.05 | 0.19 |
| Lateral Ventral PFC | | -0.35 | -0.20 | -0.24 |
| Cingulate Posterior | | 0.11 | -0.04 | 0.08 |
| Precuneus PCC | | 0.24 | -0.07 | 0.09 |
| Medial PFC | | 0.02 | -0.07 | -0.10 |
| Ventral PFC | | 0.14 | 0.14 | 0.07 |
| Retrosplenial | | 0.16 | 0.05 | 0.23 |
| Parahippocampal Cortex | | 0.34 | 0.35 | 0.27 |
| Temporal Parietal | | 0.16 | 0.27 | 0.01 |
| Precentral | | -0.09 | 0.21 | 0.0002 |
| Frontal Medial | | 0.26 | 0.16 | 0.18 |
| Anterior Temporal | | -0.17 | -0.13 | -0.19 |
| Testosterone: pg/mL, Estradiol: pg/mL, Cortisol: ug/dL  Abbreviations: GMV = Gray Matter Volume, PFC = Prefrontal Cortex, PCC = Posterior Cingulate Cortex | | | | |
