# Supplementary material for: Circadian Rhythms Tied to Changes in Brain Morphology in a Densely Sampled Male
Source: J Neurosci. 2024 Aug 15;44(38):e0573242024. doi: 10.1523/JNEUROSCI.0573-24.2024 (PMC11411591; doi:10.1523/JNEUROSCI.0573-24.2024)
Supplement: Table 1-3 — Correlations between cortical thickness in cortical regions and steroid hormones. Download Table 1-3, DOCX file. [file jneuro-44-e0573242024-s007.docx]

| Table 1-3. Correlations between cortical thickness in cortical regions and steroid hormones | | | |
| --- | --- | --- | --- |
|  | Correlation | | |
| Brain Region | Testosterone (saliva) | Estradiol (serum) | Cortisol  (saliva) |
| Extrastriate Cortex | 0.52** | 0.59** | 0.45** |
| Striate Cortex | 0.61*** | 0.66*** | 0.65*** |
| Extrastriate Inferior | 0.43* | 0.53** | 0.49** |
| Striate Calcerine | 0.61*** | 0.68*** | 0.71*** |
| Extrastriate Superior | 0.63*** | 0.58** | 0.58*** |
| Somatomotor | 0.42* | 0.21 | 0.32 |
| Auditory | 0.26 | 0.06 | 0.18 |
| Insula | 0.09 | -0.007 | -0.01 |
| Secondary Somatomotor | 0.16 | 0.003 | 0.13 |
| Central | 0.31 | 0.11 | 0.29 |
| Temporal Occipital | 0.45** | 0.39 | 0.34 |
| Parietal Occipital | 0.45** | 0.25 | 0.36* |
| Superior Parietal Lobule | 0.36* | 0.10 | 0.30 |
| Post Central | 0.27 | -0.11 | 0.13 |
| Frontal Eye Fields | 0.21 | -0.09 | 0.04 |
| Precentral Ventral | 0.39* | 0.10 | 0.38* |
| Parietal Operculum | 0.35* | 0.05 | 0.20 |
| Frontal Operculum | 0.07 | -0.21 | -0.06 |
| Parietal Medial | 0.31 | 0.19 | 0.30 |
| Lateral PFC | 0.06 | -0.19 | 0.05 |
| Orbitofrontal Cortex | -0.22 | -0.13 | -0.26 |
| Medial Posterior PFC | 0.30 | 0.27 | 0.33 |
| Temporal Pole | 0.13 | 0.15 | 0.09 |
| Temporal | 0.29 | 0.23 | 0.27 |
| Intraparietal Sulcus | 0.34 | 0.11 | 0.32 |
| Lateral Dorsal PFC | -0.11 | -0.30 | -0.10 |
| Mid-Cingulate | -0.07 | -0.32 | -0.06 |
| Inferior Parietal Lobule | 0.34 | 0.11 | 0.30 |
| Dorsal PFC | 0.07 | -0.19 | 0.08 |
| Lateral Ventral PFC | -0.22 | -0.41* | -0.29 |
| Precuneus | 0.42* | 0.29 | 0.43* |
| Cingulate Posterior | 0.69*** | 0.54** | 0.65*** |
| Precuneus PCC | 0.51** | 0.37 | 0.47** |
| Medial PFC | -0.07 | -0.26 | -0.12 |
| Ventral PFC | -0.26 | -0.37 | -0.38* |
| Retrosplenial | 0.54*** | 0.52** | 0.55*** |
| Parahippocampal Cortex | 0.38* | 0.43* | 0.32 |
| Temporal Parietal | 0.28 | 0.02 | 0.22 |
| Precentral | -0.05 | -0.17 | -0.03 |
| Frontal Medial | 0.12 | -0.09 | 0.15 |
| Anterior Temporal | -0.25 | -0.11 | -0.23 |
| FDR-corrected at *q* < .05: **p* < .05, ***p* < .01, ****p* < .001, Testosterone: pg/mL, Estradiol: pg/mL, Cortisol: ug/dL  Abbreviations: PFC = Prefrontal Cortex, PCC = Posterior Cingulate Cortex | | | |
