# Supplementary material for: Circadian Rhythms Tied to Changes in Brain Morphology in a Densely Sampled Male
Source: J Neurosci. 2024 Aug 15;44(38):e0573242024. doi: 10.1523/JNEUROSCI.0573-24.2024 (PMC11411591; doi:10.1523/JNEUROSCI.0573-24.2024)
Supplement: Table 2-1 — Global brain morphology by time of day and association with steroid hormones. Download Table 2-1, DOCX file. [file jneuro-44-e0573242024-s008.docx]

| Table 2-1: Global brain morphology by time of day and association with steroid hormones | | | | | | | |
| --- | --- | --- | --- | --- | --- | --- | --- |
| Brain Region (mm^3^) | Morning | Evening | Effect Size | p-value | Correlation |  |  |
|  | Mean (SD) | Mean (SD) | (Cohen’s d) |  | Testosterone (saliva) | Estradiol (serum) | Cortisol (saliva) |
| Total Brain Volume | 1521915.50 (853.00) | 1519316.32  (1406.86) | -2.23 | 1.19e-07^§^ | 0.58*** | 0.70*** | 0.56*** |
| Gray Matter Volume | 612900.20 (1523.69) | 609097.74  (1752.32) | -2.32 | 1.80e-08^§^ | 0.67*** | 0.69*** | 0.61*** |
| White Matter Volume | 422086.75 (1399.77) | 420897.95  (1764.38) | -0.75 | 0.026 | 0.22 | 0.41* | 0.26 |
| Cortical Thickness | 2465661.00 (30968.59) | 2421545.79  (31918.16) | -1.40 | 9.57e-05^§^ | 0.53** | 0.46* | 0.47** |
| Cerebrospinal Fluid | 884.54  (19.19) | 909.64  (24.67) | 1.14 | 0.0012^†^ | -0.51** | -0.49* | -0.37* |
| Left Lateral Ventricle | 4885.37  (58.13) | 4969.92  (75.08) | 1.26 | 0.0004^‡^ | -0.42* | -0.64*** | -0.25 |
| Right Lateral Ventricle | 4494.83  (57.22) | 4568.49  (77.41) | 1.08 | 0.0019^†^ | -0.36* | -0.52** | -0.22 |
| Third Ventricle | 931.12  (14.75) | 926.81  (15.39) | -0.29 | 0.378 | 0.34* | 0.11 | 0.28 |
| Fourth Ventricle | 2839.56  (20.50) | 2874.48  (37.24) | 1.16 | 0.0012^†^ | -0.39* | -0.56** | -0.22 |
| T-tests Bonferroni-corrected at: † *p* < .005555556 , ‡ *p* < .001111111, § *p* < .0001111111  Correlations FDR-corrected at *q* < .05: **p* < .05, ***p* < .01, ****p* < .001. Testosterone: pg/mL, Estradiol: pg/mL, Cortisol: ug/dL | | | | | | | |
