# Supplementary figures and images for: Circadian Rhythms Tied to Changes in Brain Morphology in a Densely Sampled Male
Source: J Neurosci. 2024 Aug 15;44(38):e0573242024. doi: 10.1523/JNEUROSCI.0573-24.2024 (PMC11411591; doi:10.1523/JNEUROSCI.0573-24.2024)

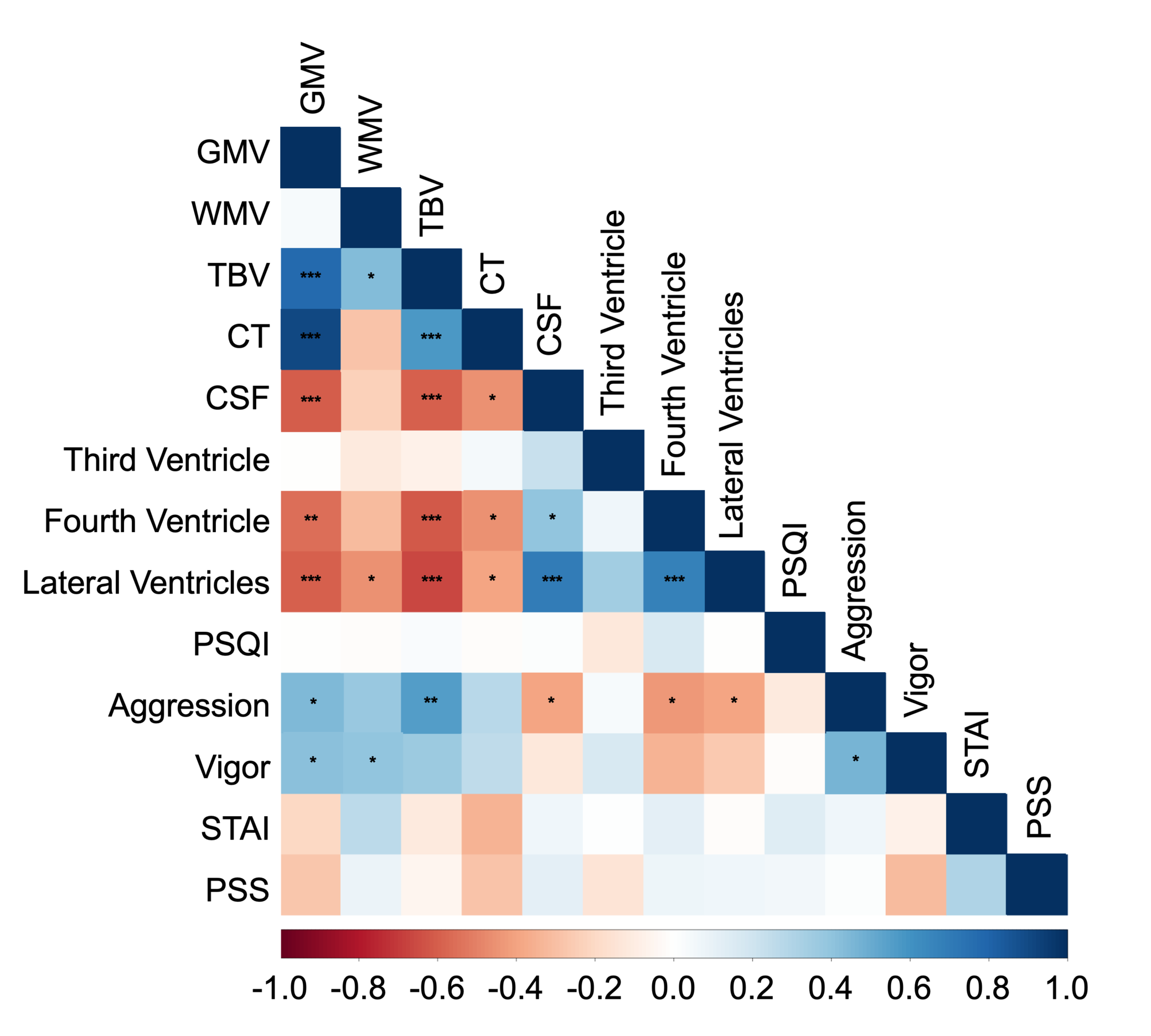

Supplement: Figure 2-1 — Correlations between global brain morphology and behavioral assessments. Correlation plot shows relationships between global brain morphology measures and mood assessments. Cool colors indicate positive correlations and warm colors indicate negative correlations. FDR-corrected at q < .05: *p < .05, **p < .01, ***p < .001Abbreviations: GMV = Gray Matter Volume; WMV = White Matter Volume; TBV = Total Brain Volume; CT = Cortical Thickness, CSF = Cerebrospinal Fluid; PSQI = Pittsburgh Sleep Quality Index; STAI = State-Trait Anxiety Inventory; PSS = Perceived Stress Scale. Download Figure 2-1, TIF file. [file jneuro-44-e0573242024-s001.tif]

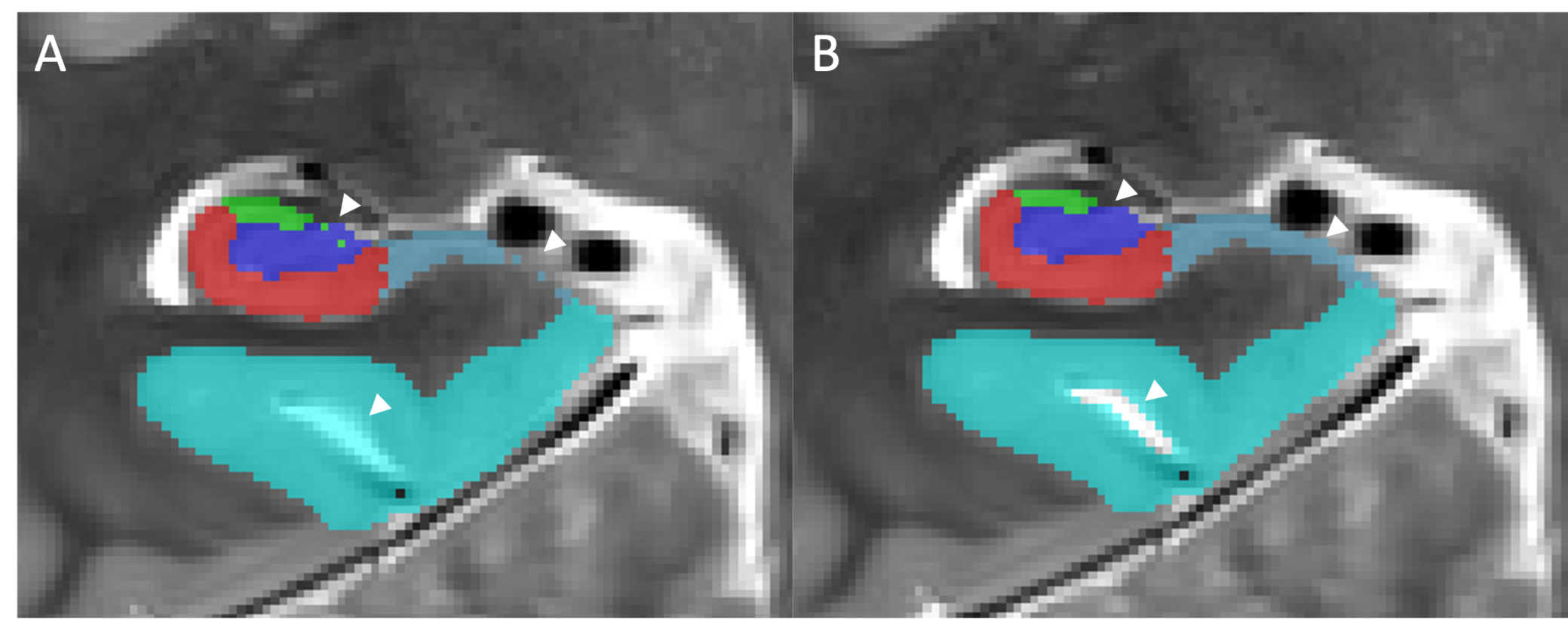

Supplement: Figure 4-3 — Manual segmentation of medial temporal lobe. Example of manual retouching. A) Sample original ASHS segmentation with erroneous labeling of CA2/3 within dentate gyrus, missing/unlabeled voxels within the subiculum, and inclusion of CSF in PHC label. B) Segmentation of CA2/3, subiculum and PHC after manual retouching. Download Figure 4-3, TIF file. [file jneuro-44-e0573242024-s002.tif]
