# Supplementary material for: Circadian Rhythms Tied to Changes in Brain Morphology in a Densely Sampled Male
Source: J Neurosci. 2024 Aug 15;44(38):e0573242024. doi: 10.1523/JNEUROSCI.0573-24.2024 (PMC11411591; doi:10.1523/JNEUROSCI.0573-24.2024)
Supplement: Table 3-1 — Gray matter volume in cortical regions by time of day. Download Table 3-1, DOCX file. [file jneuro-44-e0573242024-s004.docx]

| Table 3-1. Gray matter volume in cortical regions by time of day | | | | |
| --- | --- | --- | --- | --- |
| Brain Region (mm^3^) | Morning  Mean (SD) | Evening  Mean (SD) | Effect Size  (Cohen’s d) | p-value |
| Extrastriate Cortex | 0.747 (0.003) | 0.741 (0.003) | -2.320 | 1.36e-08*** |
| Striate Cortex | 0.853 (0.009) | 0.817 (0.010) | -3.700 | 1.20e-13*** |
| Extrastriate Inferior | 0.747 (0.007) | 0.742 (0.006) | -0.879 | 0.009 |
| Striate Calcerine | 0.801 (0.005) | 0.790 (0.006) | -2.075 | 2.01e-07*** |
| Extrastriate Superior | 0.747 (0.004) | 0.742 (0.004) | -1.330 | 0.0002** |
| Somatomotor | 0.676 (0.004) | 0.674 (0.004) | -0.492 | 0.133 |
| Auditory | 0.746 (0.003) | 0.744 (0.003) | -0.847 | 0.012 |
| Insula | 0.808 (0.003) | 0.807 (0.002) | -0.571 | 0.083 |
| Secondary Somatomotor | 0.770 (0.003) | 0.769 (0.003) | -0.344 | 0.291 |
| Central | 0.711 (0.003) | 0.710 (0.004) | -0.526 | 0.110 |
| Temporal Occipital | 0.818 (0.003) | 0.812 (0.003) | -1.719 | 4.72e-06*** |
| Parietal Occipital | 0.740 (0.003) | 0.738 (0.002) | -1.010 | 0.003 |
| Superior Parietal Lobule | 0.661 (0.003) | 0.662 (0.003) | 0.246 | 0.447 |
| Post Central | 0.667 (0.004) | 0.666 (0.003) | -0.186 | 0.564 |
| Frontal Eye Fields | 0.744 (0.006) | 0.742 (0.003) | -0.403 | 0.216 |
| Precentral Ventral | 0.769 (0.006) | 0.764 (0.004) | -1.029 | 0.003 |
| Parietal Operculum | 0.732 (0.004) | 0.731 (0.003) | -0.537 | 0.102 |
| Frontal Operculum | 0.759 (0.003) | 0.757 (0.004) | -0.543 | 0.099 |
| Parietal Medial | 0.705 (0.003) | 0.704 (0.003) | -0.363 | 0.264 |
| Lateral PFC | 0.714 (0.002) | 0.712 (0.002) | -0.987 | 0.004 |
| Orbitofrontal Cortex | 0.747 (0.003) | 0.746 (0.004) | -0.321 | 0.324 |
| Medial Posterior PFC | 0.733 (0.004) | 0.731 (0.004) | -0.430 | 0.187 |
| Temporal Pole | 0.797 (0.004) | 0.795 (0.004) | -0.481 | 0.142 |
| Temporal | 0.784 (0.002) | 0.783 (0.002) | -0.499 | 0.128 |
| Intraparietal Sulcus | 0.683 (0.003) | 0.682 (0.003) | -0.422 | 0.195 |
| Lateral Dorsal PFC | 0.691 (0.004) | 0.688 (0.003) | -0.786 | 0.019 |
| Mid-Cingulate | 0.665 (0.008) | 0.664 (0.007) | -0.150 | 0.642 |
| Inferior Parietal Lobule | 0.688 (0.002) | 0.686 (0.002) | -0.745 | 0.026 |
| Dorsal PFC | 0.701 (0.004) | 0.698 (0.004) | -0.581 | 0.078 |
| Lateral Ventral PFC | 0.731 (0.002) | 0.732 (0.002) | 0.468 | 0.154 |
| Precuneus | 0.757 (0.003) | 0.754 (0.003) | -1.154 | 0.0009* |
| Cingulate Posterior | 0.651 (0.008) | 0.651 (0.005) | -0.017 | 0.958 |
| Precuneus PCC | 0.787 (0.003) | 0.786 (0.002) | -0.343 | 0.290 |
| Medial PFC | 0.737 (0.003) | 0.737 (0.002) | 0.057 | 0.859 |
| Ventral PFC | 0.744 (0.003) | 0.744 (0.003) | -0.257 | 0.427 |
| Retrosplenial | 0.721 (0.005) | 0.719 (0.006) | -0.239 | 0.463 |
| Parahippocampal Cortex | 0.745 (0.005) | 0.741 (0.005) | -0.778 | 0.020 |
| Temporal Parietal | 0.748 (0.002) | 0.747 (0.003) | -0.383 | 0.241 |
| Precentral | 0.677 (0.005) | 0.677 (0.004) | 0.014 | 0.965 |
| Frontal Medial | 0.694 (0.005) | 0.692 (0.003) | -0.342 | 0.291 |
| Anterior Temporal | 0.777 (0.004) | 0.778 (0.006) | 0.274 | 0.402 |
| Bonferroni-corrected at **p <* . 001219512, ***p < .* 0002439024*, ***p <* 2.439024e-05  Abbreviations: PFC = Prefrontal Cortex, PCC = Posterior Cingulate Cortex | | | | |
