# Supplementary material for: Circadian Rhythms Tied to Changes in Brain Morphology in a Densely Sampled Male
Source: J Neurosci. 2024 Aug 15;44(38):e0573242024. doi: 10.1523/JNEUROSCI.0573-24.2024 (PMC11411591; doi:10.1523/JNEUROSCI.0573-24.2024)
Supplement: Table 3-2 — Cortical thickness in cortical regions by time of day. Download Table 3-2, DOCX file. [file jneuro-44-e0573242024-s005.docx]

| Table 3-2. Cortical thickness in cortical regions by time of day | | | | |
| --- | --- | --- | --- | --- |
| Brain Region (mm^3^) | Morning  Mean (SD) | Evening  Mean (SD) | Effect Size  (Cohen’s d) | p-value |
| Extrastriate Cortex | 3.603 (0.061) | 3.507 (0.066) | -1.510 | 3.57e-05** |
| Striate Cortex | 4.948 (0.182) | 4.542 (0.173) | -2.284 | 1.88e-08*** |
| Extrastriate Inferior | 3.859 (0.135) | 3.720 (0.094) | -1.199 | 0.0006* |
| Striate Calcerine | 3.080 (0.098) | 2.851 (0.096) | -2.360 | 9.25e-09*** |
| Extrastriate Superior | 2.751 (0.051) | 2.659 (0.059) | -1.663 | 8.83e-06*** |
| Somatomotor | 1.852 (0.029) | 1.820 (0.039) | -0.930 | 0.007 |
| Auditory | 2.745 (0.050) | 2.719 (0.044) | -0.552 | 0.093 |
| Insula | 4.024 (0.066) | 4.022 (0.053) | -0.045 | 0.888 |
| Secondary Somatomotor | 3.051 (0.044) | 3.033 (0.045) | -0.414 | 0.205 |
| Central | 1.975 (0.037) | 1.951 (0.031) | -0.709 | 0.033 |
| Temporal Occipital | 4.661 (0.074) | 4.592 (0.085) | -0.868 | 0.010 |
| Parietal Occipital | 3.314 (0.092) | 3.240 (0.077) | -0.860 | 0.011 |
| Superior Parietal Lobule | 1.934 (0.043) | 1.898 (0.033) | -0.937 | 0.006 |
| Post Central | 1.981 (0.058) | 1.960 (0.052) | -0.383 | 0.239 |
| Frontal Eye Fields | 2.323 (0.046) | 2.310 (0.045) | -0.287 | 0.376 |
| Precentral Ventral | 2.790 (0.069) | 2.745 (0.049) | -0.753 | 0.024 |
| Parietal Operculum | 2.657 (0.054) | 2.626 (0.055) | -0.570 | 0.083 |
| Frontal Operculum | 2.711 (0.045) | 2.714 (0.037) | 0.059 | 0.856 |
| Parietal Medial | 2.441 (0.052) | 2.405 (0.037) | -0.775 | 0.021 |
| Lateral PFC | 2.243 (0.035) | 2.243 (0.040) | 0.012 | 0.970 |
| Orbitofrontal Cortex | 3.472 (0.045) | 3.493 (0.038) | 0.516 | 0.115 |
| Medial Posterior PFC | 2.980 (0.041) | 2.951 (0.032) | -0.779 | 0.020 |
| Temporal Pole | 4.135 (0.065) | 4.123 (0.058) | -0.197 | 0.542 |
| Temporal | 3.873 (0.056) | 3.840 (0.052) | -0.617 | 0.062 |
| Intraparietal Sulcus | 2.266 (0.080) | 2.214 (0.070) | -0.694 | 0.036 |
| Lateral Dorsal PFC | 2.175 (0.039) | 2.185 (0.037) | 0.255 | 0.431 |
| Mid-Cingulate | 1.877 (0.034) | 1.884 (0.027) | 0.215 | 0.506 |
| Inferior Parietal Lobule | 2.817 (0.074) | 2.767 (0.065) | -0.713 | 0.032 |
| Dorsal PFC | 2.207 (0.031) | 2.205 (0.035) | -0.055 | 0.865 |
| Lateral Ventral PFC | 3.075 (0.048) | 3.111 (0.041) | 0.788 | 0.018 |
| Precuneus | 2.763 (0.068) | 2.699 (0.064) | -0.960 | 0.005 |
| Cingulate Posterior | 2.265 (0.071) | 2.154 (0.054) | -1.773 | 2.93e-06*** |
| Precuneus PCC | 3.991 (0.059) | 3.934 (0.045) | -1.092 | 0.002 |
| Medial PFC | 3.263 (0.038) | 3.278 (0.049) | 0.342 | 0.295 |
| Ventral PFC | 2.776 (0.036) | 2.799 (0.027) | 0.720 | 0.030 |
| Retrosplenial | 2.964 (0.063) | 2.877 (0.052) | -1.515 | 3.27e-05** |
| Parahippocampal Cortex | 3.567 (0.060) | 3.528 (0.049) | -0.711 | 0.032 |
| Temporal Parietal | 3.250 (0.059) | 3.221 (0.043) | -0.551 | 0.093 |
| Precentral | 1.975 (0.038) | 1.979 (0.035) | 0.100 | 0.755 |
| Frontal Medial | 2.468 (0.041) | 2.460 (0.044) | -0.199 | 0.538 |
| Anterior Temporal | 3.994 (0.102) | 4.032 (0.093) | 0.392 | 0.228 |
| Bonferroni-corrected at **p <* . 001219512, ***p < .* 0002439024*, ***p <* 2.439024e-05  Abbreviations: PFC = Prefrontal Cortex, PCC = Posterior Cingulate Cortex | | | | |
