# Supplementary material for: Circadian Rhythms Tied to Changes in Brain Morphology in a Densely Sampled Male
Source: J Neurosci. 2024 Aug 15;44(38):e0573242024. doi: 10.1523/JNEUROSCI.0573-24.2024 (PMC11411591; doi:10.1523/JNEUROSCI.0573-24.2024)
Supplement: Table 4-1 — Subcortical gray matter volume by time of day and association with steroid hormones. Download Table 4-1, DOCX file. [file jneuro-44-e0573242024-s009.docx]

| Table 4-1: Subcortical gray matter volume by time of day and association with steroid hormones | | | | | | | |
| --- | --- | --- | --- | --- | --- | --- | --- |
| Brain Region (mm^3^) | Morning | Evening | Effect Size | p-value | Correlation | | |
|  | Mean (SD) | Mean (SD) | Cohen’s d |  | Testosterone (saliva) | Estradiol (serum) | Cortisol (saliva) |
| Left Lateral Ventricle | 4885.36 (58.13) | 4969.92 (75.08) | 1.26 | 0.0004^†^ | -0.42 | -0.64** | -0.25 |
| Left Cerebellum- White Matter | 14015.93 (165.87) | 13939.95 (171.26) | -0.45 | 0.17 | 0.21 | 0.29 | 0.17 |
| Left Cerebellum- Cortex | 48953.44 (230.41) | 48304.45 (251.99) | -2.69 | 5.24e-10^§^ | 0.68*** | 0.72*** | 0.70*** |
| Left Thalamus | 8812.60 (49.98) | 8795.86 (77.70) | -0.26 | 0.43 | 0.04 | -0.15 | 0.02 |
| Left Caudate | 3658.14 (29.84) | 3636.28 (23.70) | -0.81 | 0.02 | 0.28 | 0.47 | 0.24 |
| Left Putamen | 6007.22 (41.23) | 5991.38 (56.31) | -0.32 | 0.33 | -0.08 | 0.12 | -0.08 |
| Left Pallidum | 2269.21 (30.90) | 2259.04 (28.22) | -0.34 | 0.29 | 0.14 | 0.35 | 0.06 |
| Third Ventricle | 931.12  (14.75) | 926.81  (15.39) | -0.29 | 0.38 | 0.34 | 0.11 | 0.28 |
| Fourth Ventricle | 2839.56 (20.50) | 2874.48 (37.24) | 1.16 | 0.0012^†^ | -0.39 | -0.56* | -0.22 |
| Brainstem | 22133.76 (64.88) | 22040.95 (83.13) | -1.24 | 0.0005^†^ | 0.37 | 0.62** | 0.32 |
| Left Hippocampus | 4332.31 (28.61) | 4323.51 (29.20) | -0.30 | 0.35 | 0.16 | 0.40 | 0.21 |
| Left Amygdala | 2058.82 (28.21) | 2045.35 (30.87) | -0.46 | 0.16 | 0.24 | 0.13 | 0.16 |
| Left Ventral Diencephalon | 4299.03 (35.10) | 4282.24 (36.70) | -0.47 | 0.15 | 0.13 | 0.35 | 0.26 |
| Right Lateral Ventricle | 4494.83 (57.22) | 4568.49 (77.41) | 1.08 | 0.002 | -0.36 | -0.52* | -0.22 |
| Right Cerebellum- White Matter | 13393.34 (164.83) | 13438.23 (152.38) | 0.28 | 0.38 | -0.06 | 0.09 | -0.12 |
| Right Cerebellum- Cortex | 51502.25 (216.14) | 51020.53 (250.68) | -2.06 | 2.06e-07^‡^ | 0.72*** | 0.58* | 0.63*** |
| Right Thalamus | 8856.40 (70.03) | 8800.67 (80.43) | -0.74 | 0.03 | 0.24 | 0.47 | 0.23 |
| Right Caudate | 3905.44 (24.04) | 3890.93 (26.21) | -0.58 | 0.08 | 0.14 | 0.09 | 0.22 |
| Right Putamen | 6081.02 (35.89) | 6059.94 (39.22) | -0.56 | 0.09 | 0.18 | 0.40 | 0.05 |
| Right Pallidum | 2197.16 (31.56) | 2208.39 (29.99) | 0.36 | 0.26 | -0.19 | -0.23 | -0.18 |
| Right Hippocampus | 4406.86 (26.96) | 4362.16 (27.04) | -1.66 | 8.49e-06^§^ | 0.55** | 0.61** | 0.44* |
| Right Amygdala | 1821.96 (26.08) | 1810.32 (22.84) | -0.47 | 0.15 | 0.13 | 0.21 | 0.20 |
| Right Ventral Diencephalon | 4447.59 (34.72) | 4450.11 (28.32) | 0.08 | 0.81 | -0.21 | -0.09 | -0.02 |
| Corpus Callosum- Posterior | 1299.56 (13.01) | 1292.95 (17.40) | -0.43 | 0.19 | 0.27 | 0.29 | 0.45* |
| Corpus Callosum- Mid Posterior | 756.93  (9.49) | 746.71  (8.49) | -1.13 | 0.0011^†^ | 0.28 | 0.42 | 0.36 |
| Corpus Callosum- Central | 789.01  (23.98) | 783.97  (26.62) | -0.20 | 0.54 | -0.12 | -0.06 | -0.004 |
| Corpus Callosum- Mid Anterior | 630.53  (5.42) | 629.68  (7.93) | -0.12 | 0.70 | -0.05 | 0.31 | -0.06 |
| Corpus Callosum- Anterior | 1208.03 (12.86) | 1209.94 (14.53) | 0.14 | 0.67 | -0.01 | -0.20 | -0.14 |
| T-tests Bonferroni-corrected at: † *p* < 0.001785714, ‡ *p* < 0.0003571429, § *p* < 3.571429e-05  Correlations FDR-corrected at *q* < .05: **p* < .05, ***p* < .01, ****p* < .001, Testosterone: pg/mL, Estradiol: pg/mL, Cortisol: ug/dL | | | | | | | |
