# Supplementary material for: Circadian Rhythms Tied to Changes in Brain Morphology in a Densely Sampled Male
Source: J Neurosci. 2024 Aug 15;44(38):e0573242024. doi: 10.1523/JNEUROSCI.0573-24.2024 (PMC11411591; doi:10.1523/JNEUROSCI.0573-24.2024)
Supplement: Table 4-2 — Medial temporal lobe gray matter volume by time of day and association with steroid hormones. Download Table 4-2, DOCX file. [file jneuro-44-e0573242024-s010.docx]

| Table 4-2: Medial temporal lobe gray matter volume by time of day and association with steroid hormones | | | | | | | | | | | | | |  |
| --- | --- | --- | --- | --- | --- | --- | --- | --- | --- | --- | --- | --- | --- | --- |
| Brain Region (mm^3^) | Morning | | Evening | | Effect Size | | p-value | | Correlation | | | | | |
|  | Mean (SD) | | Mean (SD) | | Cohen’s d | |  | | Testosterone  (saliva) | | Estradiol (serum) | | Cortisol  (saliva) | |
| CA1 | 572.28  (21.92) | 563.57  (10.92) | | -0.50 | | 0.12 | | 0.04 | | 0.23 | | 0.14 | |  |
| CA2/3 | 152.59  (14.34) | 154.80  (9.79) | | 0.18 | | 0.58 | | 0.12 | | -0.08 | | -0.17 | |  |
| Dentate Gyrus | 547.18  (30.56) | 536.58  (16.37) | | -0.43 | | 0.18 | | 0.27 | | 0.18 | | 0.07 | |  |
| Entorhinal Cortex | 646.19  (26.68) | 641.33  (24.38) | | -0.19 | | 0.56 | | 0.14 | | 0.22 | | 0.01 | |  |
| Parahippocampal Cortex | 3079.51 (87.53) | 3078.87  (93.57) | | -0.01 | | 0.98 | | -0.07 | | 0.05 | | 0.05 | |  |
| Perirhinal Cortex | 2298.65  (79.64) | 2295.97  (87.23) | | -0.03 | | 0.92 | | 0.04 | | -0.11 | | 0.13 | |  |
| Subiculum | 357.58  (16.68) | 349.27  (15.21) | | -0.52 | | 0.11 | | 0.30 | | 0.10 | | 0.35 | |  |
| Testosterone: pg/mL, Estradiol: pg/mL, Cortisol: ug/dL | | | | | | | | | | | | | |  |
